# Hereditary Insanity

**Published:** 1896-12

**Authors:** 


For the Texas Medical Journal.

### HEREDITARY INSANITY.

TO THOSE who are accustomed to read the able defense in many of our celebrated murder trials, as well as those who have extensive experience in the treatment of the insane, the question as to how far heredity goes, as the cause of the various forms and phases of mental aberration, is an interesting one.

It is usual for cases to fall in the hands of the alienist with but little or no authentic family history, and in the absence of such knowledge, those in charge of such cases, in assigning a cause, usually ascribe it to some physical derangement, or to causes acting on the emotional nature, such as loss of property, the death of dear relatives or friends, or to intemperance or religious excitement. It is believed that the usual, apparent, frivilous causes given, if acting on a normally healthy brain, would have produced no such serious results; but back of this there must have been some latent predominating cause.

To what extent does the influence of mental heredity affect the mental processes and control the emotional nature?

It is a fact beyond question, that parential influences are active and potential agents in determining the physical conformation of offspring.

Organic diseases of the brain, leading to irregularity of development, which, functionally, may manifest themselves in insanity; these and other laws of development, occurring in a degenerating family line, often traceable through more than one generation, furnish valuable arguments by analogy in support of the theories of heredity as a predisposing cause in most of the forms of mental disease.

"Individuals are sometimes born with so defective nervous organization, that as soon as the first manifestations of higher mental life appear, or should appear, the defective or perverted anatomical foundation exhibits its nefarious influence in the defective or perverted state of the mind."—Spitzka.

It is contended by some, that the influence of heredity is confind to those forms of insanity which we have been able, only, to account for by abnormal brain development, or by well marked cerebral defects; but there are undoubted cases of inherited origin, in which cerebral defects are not discernable.

It is noteworthy that visitors, going through wards of an insane asylum, constantly direct their attention to those patients who exhibit a well marked asymetry of cranial development; such as these, they readily agree are proper subjects of the alienist's care, but those of normal personal appearance, who are able to talk to them on many subjects, rationally, and reason coherently, though they be known as dangerous, homicidal maniacs, are suspected of being unjustly deprived of their liberty. These cases of abnormal cranial development are, of course, most often thought to be the subjects of heredity; but does not heredity also exert an influence upon those phases of mental unsoundness which have to do with those of an emotional nature, and oftentimes associated with phenomenal mental development? How much can be charged to heredity,—for instance, in those persons who, ofttimes endowed with brilliant mental activity, yet who are ludicrously mono-maniacal on some subject, or dangerously so on another? What of the so-called pyro-maniac, whose only morbid impulses are to burn and to destroy? Of the kleptomaniac who, beyond want, yet has an uncontrollable desire to steal; or to the mother or father who is seized with the fancy to sacrifice their children to appease the imaginary wrath of an offended deity, or to kill, in the most cruel and merciless manner, those who are bound to them by the strongest bonds of love and aftion? What molecular change in the brain structure, what morbid desire, what *hereditary taint intensified*, controls those who, during their earlier lives, have been surrounded with all the refining and ennobling influences of society, yet who commit crimes, so revolting in their nature, that all the world shudders?

If the sins of the father are to be "visited upon the children even unto the third and fourth generations," in a physical sense, is it not reasonable to suppose that morbid desires and perverted inclinations intensified, may produce, by inherited predisposition, a moral degeneration? This is a broad field for future cultivation, in which, up to the present, but little gleaning has been done. This is a field in which the medico-legal student can pursue his studies with infinite profit to humanity; and the day will come when the law can hold the scales of justice well balanced by the light which science can lend. W. L. B.

